# High dosage accelerated intermittent theta burst stimulation without precision targeting and dosing in depression: an open-label pilot study

**DOI:** 10.1007/s00406-025-02067-z

**Published:** 2025-07-24

**Authors:** Miaoxi Chen, Jonas Björklund, Kai-Yen Chang, Gerrit Burkhardt, Lucia Bulubas, Simone Weller, Kristin Hagenah, Daniel Kesser, Andre R. Brunoni, Frank Padberg, Ulrike Vogelmann

**Affiliations:** 1 Department of Psychiatry and Psychotherapy, Ludwig Maximilians-University Hospital, Nussbaumstr. 7, 80336 Munich, Germany; 2 International Max Planck Research School for Translational Psychiatry (IMPRS-TP), Munich, Germany; 3 Deutsches Zentrum für Psychische Gesundheit (DZPG), Partner Site, Munich-Augsburg, Germany; 4 Department of Psychiatry and Psychotherapy, Neurophysiology and Interventional Neuropsychiatry, University Hospital Tuebingen, Tuebingen, Germany; 5 Deutsches Zentrum Für Psychische Gesundheit (DZPG), Partner Site, Tübingen, Germany; 6 Service of Interdisciplinary Neuromodulation, Department and Institute of Psychiatry, University of São Paulo Medical School, São Paulo, Brazil; 7 Department of Psychiatry and Psychotherapy, School of Medicine, Technical University of Munich (TUM), Munich, Germany; 8 Laboratory of Neuroscience and National Institute of Biomarkers in Psychiatry, Department and Institute of Psychiatry, University of São Paulo Medical School, São Paulo, Brazil

**Keywords:** Treatment-resistant depression, rTMS, aiTBS, Antidepressant effect

## Abstract

**Background:**

High dosage accelerated intermittent theta-burst stimulation (aiTBS) protocols (10 sessions per day for 5 days) combined with precision targeting and depth adjusted iTBS intensity yield high response and remission rates in depression. However, disentangling their efficacy components to develop pragmatic mental health solutions is challenging. This pilot study applied such a high dosage aiTBS protocol without using any precision features.

**Methods:**

Eight patients with treatment-resistant depression (TRD) underwent open-label aiTBS targeting the left dorsolateral prefrontal cortex (DLPFC) using the Beam F3 algorithm. Over 5 days, patients received 50 aiTBS sessions, each delivering 1800 pulses at 90% resting motor threshold with 50-min inter-session intervals. All patients underwent a 4 weeks follow-up without stimulation, were offered tDCS for 4 weeks thereafter and had a final follow-up after 6 months. Treatment effects were assessed by clinical and cognitive measures.

**Results:**

Patients received 46 aiTBS sessions on average. At one-month follow-up, mean MADRS scores decreased by -12.50 ± 9.81 (Cohen’s d = 2.83; 95% CI, 2.34–3.32; p < 0.001), with response and remission rates of 50% and 12.5%, respectively. After tDCS, 28.6% and 14.3% sustained response and remission, which declined to 16.7% and 0% at six months.

**Conclusion:**

This pilot trial evidenced the antidepressant effect of a high dosage aiTBS protocol comparable with the Stanford Neuromodulation Therapy (SNT) approach but without individualized precision components. Its effectiveness appeared lower than previously reported for SNT. Randomized controlled trials should systematically investigate the contribution of precision components to the overall effectiveness of aiTBS in depression.

This trial is a part of a real-world clinical study of non-invasive brain stimulation treatments conducted at our department (preregistered at DRKS-ID: DRKS00024776, drks.de).

**Supplementary Information:**

The online version contains supplementary material available at 10.1007/s00406-025-02067-z.

## Introduction

Repetitive transcranial magnetic stimulation (rTMS) of the dorsolateral prefrontal cortex (DLPFC) is widely used for treating depressive disorders and other syndromes. [1] However, there is further need for developing efficacious treatment strategies for people with depression, who do not sufficiently benefit from antidepressant pharmacotherapy, psychological interventions and standard rTMS protocols. In this respect, the field of non-invasive brain stimulation (NIBS) is moving fast from conventional rTMS protocols [2, 3] to personalized approaches, e.g. guided by functional connectivity magnetic resonance imaging (fcMRI) for individual targeting and dosing [4, 5] or potentially faster acting accelerated rTMS (arTMS) which aims to enhance response rates and shorten the time to response by applying more than one rTMS session per day [6, 7].

While a randomized clinical trial (RCT) showing the non-inferiority of intermittent TBS (iTBS) to a standard 10 Hz rTMS in ameliorating depressive symptoms [2, 3], iTBS has also been selected as a promising protocol for arTMS, and accelerated iTBS (aiTBS) has been proposed as novel rTMS strategy. Whereas a meta-analysis including 239 participants from five RCTs found modest overall efficacy of aiTBS with considerable heterogeneity in parameters and protocols [8], a very recent RCT by Ramos et al. [9] applied a pragmatic aiTBS protocol with three sessions per day in patients with TRD. The study showed that aiTBS significantly reduced depressive symptoms compared to sham stimulation, highlighting its potential as an effective treatment option.

There is one approach spearheading this development towards more efficacious and faster acting rTMS methods, i.e. the Stanford Neuromodulation Therapy (SNT) approach. SNT is an aiTBS protocol which combines both high dosage iTBS as well as individual functional and structural MRI based targeting. In the SNT protocol, 10 iTBS sessions (each extended to 1800 stimuli) per day, spaced by 50 min intervals, are applied for five days. Moreover, the SNT approach includes important features of precision targeting (i.e. with a personalized fMRI connectivity base target) and dosing (i.e. coil-cortex distance adapted stimulation intensity). It has shown potential for both rapid and high response [10, 11]. The first open-label trial showed response and remission rates of 70% and 60%, respectively, four weeks after the one week aiTBS treatment [10]. The subsequent sham-controlled RCT confirmed these findings with impressive cumulative response and remission rates of 85.7% and 78.6%, respectively, at the same time point [11].

To date, it is unclear which of the three components of the SNT approach (i.e. 10 extended iTBS sessions, fcMRI based targeting and personalized iTBS stimulation intensity) contributes to this high efficacy. Large RCTs with multiple arms would be needed to replicate the findings of the first RCT [11]. We therefore investigated in a first step, how a high intensity aiTBS protocol with the same characteristics as the SNT approach performs in an open-label clinical study. Besides using similar outcome measures, we have added a follow-up period of 6 months with a one month period of transcranial direct current stimulation (tDCS) at home as previously reported to be effective as antidepressant intervention [12].

## Methods

### Trial design

This open-label trial was conducted in an outpatient setting in the Department of Psychiatry and Psychotherapy at the LMU University Hospital with enrollment from October 2023 through April 2024. We included TRD patients with moderate to severe depressive symptoms (17-item Hamilton Depression Scale [HAM-D] scores >  = 20). Treatment resistance was defined as non-response to at least one adequate antidepressant medication assessed by the Antidepressant Treatment History Form: Short Form (ATHF-SF) during the current episode. We did not include patients with addiction disorder within the last 2 years, personality disorder, psychotic symptoms, brain pathologies indicated by structural MRI (sMRI), a history of epileptic seizures, or any unstable or insufficient treatment (for example insufficiently treated hypertensive crises or angina pectoris symptoms). Our trial was approved by the LMU local ethic board and conducted in accordance with the Declaration of Helsinki. All patients signed written informed consent. This trial is a part of a real-world clinical study of non-invasive brain stimulation treatments conducted at our department (preregistered at DRKS-ID: DRKS00024776, drks.de).

### Interventions

**Fig. 1:**
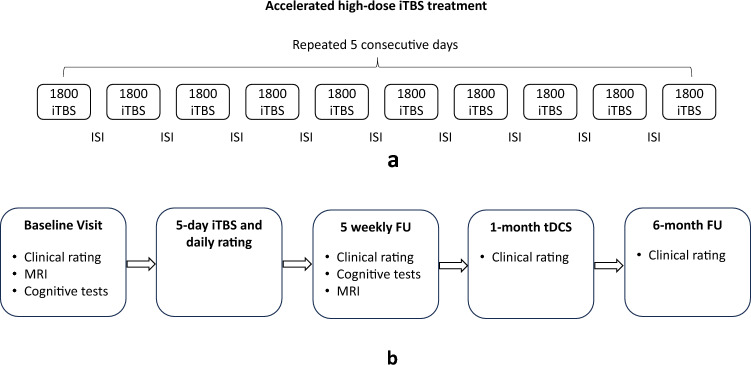
Study design. Panel a presents the daily stimulation regime and intermittent theta-burst stimulation pattern for each participant. Panel b represents the entire study flow. Clinical rating includes MADRS, 17-item HAM-D and BDI-II, daily rating was assessed by 6-item HAM-D. ISI = intersession interval; FU = follow-up

A PowerMAG clinical 100 system (Mag & More, Munich, Germany) was used to deliver iTBS treatment (aCool coil and PowerMag Stimulator); 60 cycles of 10 bursts consisting of three pulses at 50 Hz were delivered in 2-s trains (5 Hz) with an 8-s intertrain interval (1800 pulses per session). The accelerated high-dose iTBS treatment was applied at 90% resting motor threshold (rMT) to the left DLPFC as targeted by the Beam F3 algorithm (Beam et al., 2009; F3 measurement system, https://clinicalresearcher.org/F3/calculate.php, see Supplement Sect. 1). The stimulation was delivered for 5 consecutive days with 10 sessions daily, spaced by 50 min (Fig. 1a). After the acute aiTBS treatment, all patients underwent a follow-up period of 4 weeks, before an optional maintenance therapy with transcranial direct current stimulation (tDCS) was provided (two patients started the tDCS therapy a bit later due to personal circumstances). All patients signed the informed consent for tDCS before the first session. The maintenance treatment trial was also included in the ethics board approved trial proposal. Sooma Duo tDCS devices were loaned to the patients to conduct home-treatment (Sooma Oy, Helsinki, Finnland). A direct current of 2 mA was delivered for 30 min in each session through electrodes with a diameter of 35 cm^2^, with the anode positioned over F3, and a cathode over the F4 area using Sooma caps in three different sizes according to head circumference with the electrode placement cups already implemented. Patients were recommended to conduct tDCS at least twice a week but the actual frequency was up to their own decision (see Supplement Sect. 11, Table S5).

### Clinical assessments

Before and after both iTBS and tDCS treatments, depressive symptoms were assessed using clinician-reported (Montgomery-Åsberg Depression Rating Scale [MADRS] [13], 17-item Hamilton Depression Rating Scale [HAM-D [14, 15], 6-item HAM-D [16]) and self-reported scales (Beck Depression Inventory–II [BDI-II] [17], see Supplement Sect. 2). At the end of each day’s 10th iTBS stimulation session, depressive symptoms were assessed using the 6-item HAM-D to monitor symptom changes over time (See Supplement Sect. 2). After the iTBS treatment, the MADRS, 17-item HAM-D and BDI-II assessment was administered weekly for one month, after the tDCS maintenance treatment, and once more after 6 months to measure the effect duration (see Fig. 1b). We applied the same clinical cut-off for binary outcomes as in the SNT study [10, 11]: remission was defined as a score < 11 on the MADRS, a score < 8 on the 17-item HAM-D, a score < 5 on the 6-item HAM-D, and a score < 13 on the BDI-II; response was defined as a score reduction to baseline of at least 50% on the corresponding scales.

Apart from assessment of depressive symptoms, we also assessed cognitive functioning before and immediately after the iTBS treatment. Cognitive functioning was assessed through an established test battery (See Supplement Sect. 3).

We did not expect frequent or serious side effects considering the previous evidence [6, 10, 11]. However, unusual mental states, such as hypomania or suicidal ideation, were evaluated by a team of experienced psychiatrists at each visit. Neuroradiologists checked the sMRI before and after the aiTBS treatment.

### Statistical analyses

Statistical analyses were conducted using R (R 4.3.2, https://www.R-project.org, RStudio2023.09.1, https://www.rstudio.com). We applied linear mixed models using the lme4 package [18] and conducted post-hoc tests using the emmeans package [19]. Generalized linear mixed model was generated to assess the treatment change on MADRS, 17-item HAM-D, BDI-II and 6-item HAM-D. Fixed effects of time were assessed and random intercept at the patient level was added to regress out individual differences. All post-hoc pairwise comparisons were Bonferroni adjusted. We also analyzed item-wise changes in MADRS and 6-item HAM-D scores to better understand symptom change patterns. Due to the small sample and the uncontrolled treatment regime, outcomes related to the tDCS maintenance therapy and the 6-month follow-up were reported descriptively.

## Results

### Treatment group

Eight outpatients participated in this study (ages 23–48 years, median [IQR] age 33 [19], 5 female). All patients were outpatients with recurrent depression (current episode ≤ 2 years). The average [SD] age of depression onset was 22.63 [9.78] years with an average [SD] number of depressive episodes of 5.63 [2.97]. Baseline MADRS average [SD] score was 29.13 [3.18] and baseline 17-item HAM-D average [SD] score was 20.88 [2.67]. Participants were recruited by the study team or the regular brain stimulation outpatient service at the Center for Neuromodulation, Department of Psychiatry and Psychotherapy, LMU University Hospital. Patients who met the indication for a regular iTBS treatment as well as all inclusion criteria and were scheduled for iTBS were offered to participate in our pilot study.

All patients passed a safety evaluation for aiTBS treatment. Medication regimens remained stable at least 2 weeks before the study and throughout the aiTBS treatment phase, though adjustments were allowed during the follow-up period. Changes in both medication and non-medication treatments were documented for reference (see Supplement Sect. 5, Table S2 for detailed medication schemes).

Patients underwent 46.25 sessions on average [SD = 4.38]. All patients opted to receive tDCS maintenance therapy with the number of tDCS stimulation varying from 3 to 33 sessions over a period of 4 to 8 weeks. Table 1 summarizes patient demographics and treatment histories.

**Table 1:** Patient characteristics

Characteristic or Measure^a^ (N = 8)		
	Median	IQR
Age (years)	33	19
	**Mean**	**SD**
Age at onset of depression (years)	22.63	9.78
Education (year)	17.31	2.76
Number of depressive episodes	5.63	2.97
Number of failed antidepressant trials in the current episode (ATHF-SF)	1.63	1.51
Baseline MADRS score	29.13	3.18
Baseline 17-item HAM-D score	20.88	2.67
Baseline BDI-II score	32.63	5.27
	**N**	**%**
Female sex	5	62.50
Psychiatric comorbidity (M.I.N.I.)^b, c^	5	62.50
Number of patients with prior conventional rTMS treatment trials^d^	2	25.00
Number of patients with prior tDCS treatment trials	4	50.00
Number of patients with ECT	0	0

^a^One patient did not meet the hard 17-item HAM-D indication criterion but was recruited considering the symptom severity assessed by Montgomery-Åsberg Depression Rating Scale (MADRS score: 25) and clinical impression by our team. One other patient did not have any antidepressant drug trial during the current episode but had adequate tDCS and psychotherapy trials

^b^M.I.N.I. International Neuropsychiatric Interview

^c^Relevant comorbidities are suicidal ideation, suicidal behavior, post-traumatic stress disorder, panic disorder, generalized anxiety disorder and social phobia (details see Supplement Sect. 4, Table S1)

^d^Aside from the two patients, one additional patient attended a double-blinded bilateral TBS trial but the condition has not been revealed yet (Supplement Sect. 4, Table S1)

### General depressive symptom changes

#### Clinician reported outcomes

**Fig. 2:**
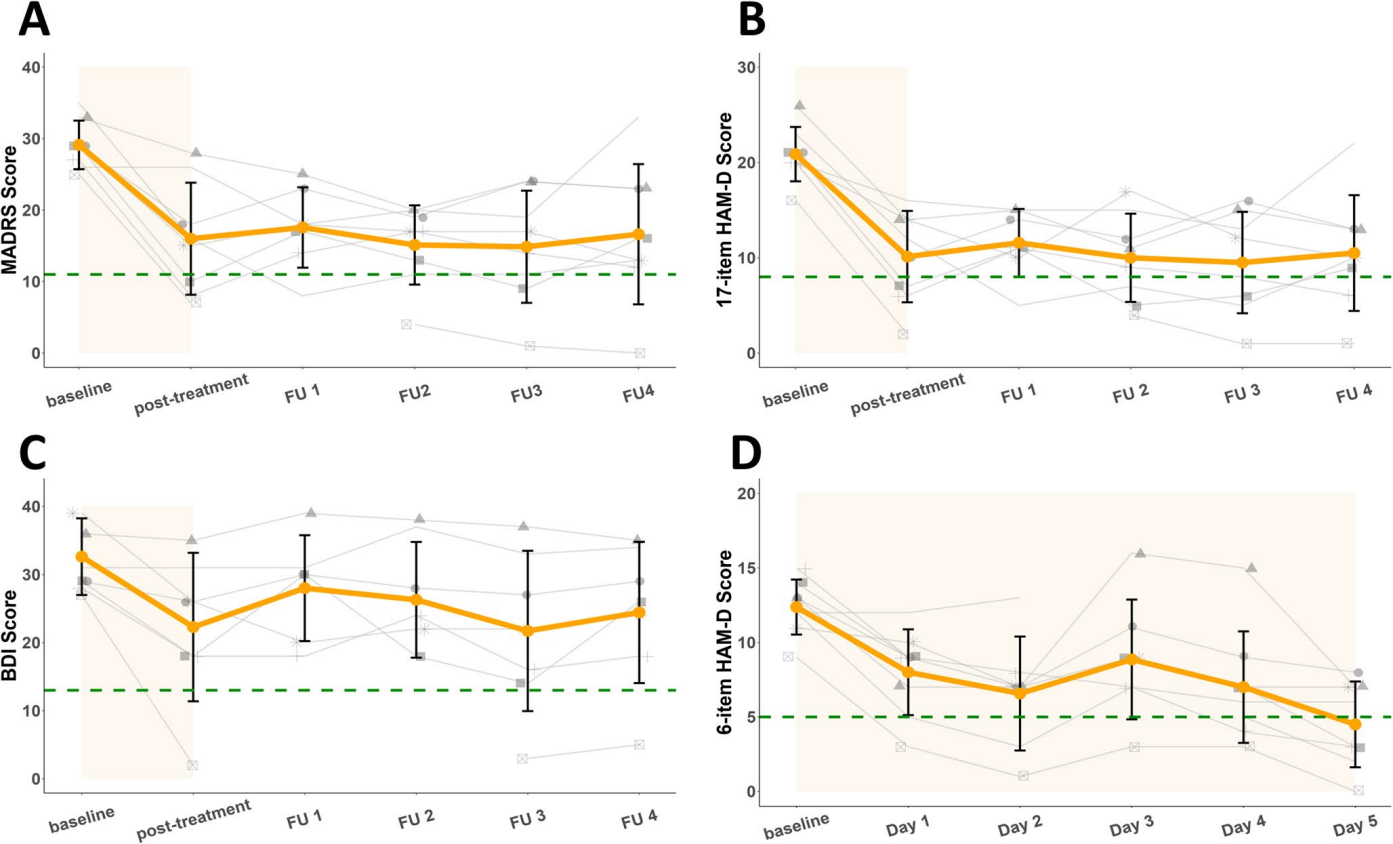
Course of mean outcome measures and individual trajectories. Panel A depicts the MADRS score change, with remission defined as a score < 11. Panel B depicts the 17-item HAM-D change, with remission defined as a score < 8. Panel C depicts the BDI-II change, with remission defined as a score < 13. Panel D depicts the daily 6-item HAM-D change, with remission defined as a score < 5. Remission thresholds are indicated by green dashed lines. Individual trajectories are represented by grey lines. The gaps in the line plots correspond to missing values, either due to missing treatment sessions or missed follow-up visits

For MADRS scores, the generalized linear mixed model revealed a significant effect of week on mean scores (F = 12.321, df = 5, p < 0.001), with all follow-up scores significantly lower than the baseline score after Bonferroni correction (p < 0.0001, Fig. 2 panel A). All participants had lower scores at post-treatment, with a mean [SD] change of − 13.13 [7.84] points (Cohen’s d = 2.97; 95% CI, 2.48 to 3.46; p < 0.001). Mean [SD] MADRS change at one-month follow-up reached -12.50 [9.81] (Cohen’s d = 2.83; 95% CI, 2.34 to 3.32; p < 0.001). MADRS response and remission rates at post-treatment were 50% and 37.5% respectively; MADRS response and remission rates at one-month follow-up were 50% and 12.5% respectively (Table 2).

**Table 2:** Change of MADRS, 17-item HAM-D, BDI-II and 6-item HAM-D scores, response and remission rates after accelerated high-dose iTBS treatment

	Immediate Post-aiTBS				One-month Post-aiTBS				One-month Post -aiTBS (cumulative outcome^a^)	
Measure	Mean (SD)	N	Response (%)	Remission (%)	Mean (SD)	N	Response (%)	Remission (%)	Response (%)	Remission (%)
MADRS	− 13.13 (7.84)	8	50.00	37.50	− 12.50 (9.81)	8	50.00	12.50	62.50	50.00
17-item HAM-D	− 10.75 (4.79)	8	62.50	37.50	− 10.38 (6.07)	8	75.00	25.00	87.50	50.00
BDI-II	− 10.34 (10.90)	7	14.29	14.29	− 8.20 (10.37)	7	14.29	14.29	37.50	12.50
6-item HAM-D	− 6.50 (2.85)	8	50.00	37.50	− 7.13 (2.66)	8	75.00	37.50	87.50	62.50

^a^Cumulative outcome is the percentage of patients who reached response or remission criteria across 4 weeks in at least one of the five post-treatment assessments (Cole et al. 2022)

For 17-item HAM-D scores, the generalized linear mixed model revealed a significant effect of week on mean scores (F = 18.43, df = 5, p < 0.001), with all follow-up scores significantly lower than the baseline score after Bonferroni correction (p < 0.0001, Fig. 2 panel B). All participants had lower scores at post-treatment, with a mean [SD] change of − 10.75 [4.79] points (Cohen’s d = 3.72; 95% CI, 3.23 to 4.21; p < 0.0001). Mean [SD] 17-item HAM-D change at one-month follow-up reached -10.38 [6.07] (Cohen’d = 3.59; 95% CI, 3.10 to 4.08 p < 0.0001). 17-item HAM-D response and remission rates at post-treatment were 62.5% and 37.5% respectively; 17-item HAM-D response and remission rates at one-month follow-up were 75% and 25%, respectively (Table 2).

To investigate acute changes resulting from the aiTBS treatment, we assessed symptoms daily using the 6-item HAM-D scale. Generalized linear mixed model revealed a significant effect of day on mean 6-item HAM-D scores (F = 9.20, df = 5, p < 0.001, Fig. 2 panel D), with all scores expect for day 3 significantly lower than the baseline score after Bonferroni correction (p < 0.05).

Item-wise changes in MADRS and 6-item HAM-D scores are detailed in Supplement Sect. 9 (Fig. S2) and Sect. 10 (Fig. S3). Notably, MADRS items “reduced sleep” and “lassitude” showed the greatest reductions, while 6-item HAM-D items “depressive mood” and “work and interests” demonstrated the largest improvements.

### Self-reported outcomes

Regarding BDI-II scores, the generalized linear mixed model revealed a significant effect of week on (F = 4.14, df = 5, p < 0.01, Fig. 2 panel C) but only post-treatment and Week 3 survive the post-hoc Bonferroni correction (p < 0.05). Average [SD] change post-treatment reached − 10.34 [10.90] points (Cohen’s d = 1.95; 95% CI, 1.43 to 2.45; p < 0.05). Average [SD] MADRS change at one-month follow-up reached -12.50 [9.81] (Cohen’s d = 2.83; 95% CI, 2.34 to 3.32; p < 0.001). BDI-II response and remission rates at post-treatment and at one-month follow-up were 14.29% and 14.29%, respectively (Table 2).

### Safety and cognitive functioning assessment

There were no serious adverse events (SAE) reported during the acute treatment and the follow-up. The most frequent side effects were fatigue and sleep disturbance (see Supplement Sect. 6, Table S3). Cognitive performance remained unchanged regarding both reaction time or accuracy on the group level.

### Explorative tDCS maintenance therapy and 6-month follow-up

All patients underwent a four-week period of tDCS treatment without any AEs. During this period, 28.57% and 14.28% of the patients sustained their response and/or remission, respectively. After six months, only 16.67% of patients were still responders, and no patient was still in remission.

Average scores, response rates and remission rates on MADRS, 17-item HAM-D and BDI scores after accelerated high-dose iTBS treatment can be found in Supplement Sect. 7, Table S4. Group change and individual change on 6-month clinical outcome can be found in Supplement Sect. 8, Fig. S1. To further examine symptom change patterns, item-wise changes for MADRS and the 6-item HAM-D are visualized in Supplement Sect. 9, Figure S2, and Sect. 10, Figure S3.

## Discussion

In this open label study, we investigated the effectiveness of a high intensity aiTBS protocol (i.e. 10 extended iTBS sessions daily with 1800 stimuli each for five days) with otherwise similar characteristics as the SNT approach apart from personalized fcMRI based targeting and dosing. Participants tolerated the treatment well. We found that this protocol led to a significant reduction of depressive symptoms within five days, which was maintained one month after treatment. Common side effects were fatigue and sleep disturbance, which were anticipated due to the long duration of this protocol. There was no change in depression scores during the tDCS treatment period.

Our aiTBS protocol achieved a 50% response rate at the one-month interval, exceeding the pooled response rate of 44.95% reported for lDLPFC iTBS (including data from the SNT RCT [11]) in the meta-analysis by Kishi et al. [20]. To date, the SNT [11] is still the most effective iTBS treatment by its high response and remission rates, 69.2% and 49.2% respectively after one month. Due to the uniqueness of the SNT protocol, we also compared our results with other accelerated rTMS protocols from RCT or cross-over design [21–23]: subjects from these studies received 5 sessions each day, 16–20 sessions in total, the stimulation target was determined by either personalized connectivity-guided target (left DLPFC—right anterior insula) or sMRI-guided target, and the sustained response rate ranges from 35 to 66%. Ramos et al. [9] applied three sessions per day with BeamF3 target positioning and achieved a significantly higher response rate in the active group (52%) compared to the sham group (22%). However, RCTs comparing aiTBS delivering no more than 3 sessions per day with standard one-session iTBS did not find significant differences and the response rates were quite low, the targeting methods ranged from the 6 cm rule, the Beam F3 method to the sMRI-guided method [24–26]. We suppose that the magnitude of the aiTBS antidepressant effect depends on a combination of factors.

One factor for the varied effect may be the stimulation dose, which involves the number of daily stimulation pulses and the number of treatment sessions. So far, only the SNT protocol has suggested a predominant dose–response relationship, other accelerated high-dose rTMS protocols, ranging broadly from 2 to 10 sessions per day, have not strongly supported this relationship. Meta-regressions from RCTs [27] found a dose–response relationship but it stagnated at some point [28, 29], whereas another meta-regression did not confirm this relationship[30]. Similar inconsistency also occurs to the number of treatment sessions [27–29, 31, 32]. The inconsistency may result from different eligible study selection criteria, for example, one meta analysis included both conventional and novel rTMS and iTBS protocols [29], whereas another included only conventional rTMS protocols [30], although the equivalency of iTBS and 10 Hz rTMS has been proved through a large-scale RCT [3]. We suggest future meta analysis include robust, comparable and extensive studies to apply generalizable reference.

Another factor might be the targeting methods and intensity correction. Neuroimaging studies found that effectiveness of rTMS for depression was related to the anticorrelation between the left DLPFC and the subgenual cingulate cortex (SGC) [33–35], therefore rsfMRI-connectivity has been analyzed to precisely target the most anticorrelated subregion of left DLPFC to SGC in the SNT trials [10, 11]. In addition to precise targeting, the SNT trials have incorporated intensity correction to account for variations in cortical depth between the individual's functional target and the primary motor cortex. This adjustment ensures that 90% rMT is consistently achieved in the intended target area. Evidence from e-field modeling also suggested significant differences in the induced E-fields at the left DLPFC between neuronavigated computational approach and BeamF3 [36]. However, no RCT has been conducted so far to directly compare those individualized computational approaches and the standard scalp-based targeting approaches. Considering the same stimulation dosage and administration with the SNT protocol [10, 11], we speculate that the discrepancy of effectiveness lies in the targeting method and individualization.

We observed an immediate drop of depressive score after the first day of aiTBS and the score continued to drop but slowlier during the rest of the week, which aligns with the open-label study from Cole et al. [10] but placebo effects due to novelty of the protocol must be considered. All of our patients either showed response directly after the treatment or did not respond at all, meaning no delayed response was observed. This was different from the SNT RCT, where several patients did not respond until the follow-up phase [11]. Comparing our results to previous studies, in which response typically occurs after three weeks [37, 38] response of our protocol occurs rapidly, probably due to high doses. Thus, accelerated high-dose iTBS protocol caters to the needs of patients who look for rapid response.

Aside from the acute effect we are also interested in the maintenance effect. Meta analysis pointed out the fading effect of rTMS and that maintenance treatment helped to sustain the antidepressant effect [39, 40]. We applied tDCS as maintenance therapy for its portability and lower cost, the antidepressant effect was quite well maintained [41]. Our results suggested a maintenance effect of tDCS, however, the causal correlation can not be concluded due to uncontrolled medication regime during follow-up and varying tDCS sessions. The antidepressant effect of tDCS is quite controversial: meta analyses showed superiority of active tDCS over sham tDCS with small to medium effect size [42–45], whereas recent large-scale sham-controlled trials did not support this evidence [46, 47]. More well-designed studies are needed to unravel the inconsistent evidence and define the responding population to tDCS.

We observed some outcome differences between clinician- and self-reported scales. BDI-II score changes did not show the comparable level of symptom reduction compared to MADRS and 17-item HAM-D score changes. We are not clear about the underlying reasons, but one factor could be maladaptive personality characteristics of patients. Some studies found BDI is more sensitive to personality characteristics whereas external rating scales focus more on depressive symptoms [48, 49]. Another factor could be different perspectives from clinicians and patients. Thus, clinician- and self-reported ratings are complementary, highlighting the importance of using multiple measures to quantify depression in future studies.

Our study has several limitations: the small sample and the open-label design made our study statistically underpowered. High placebo effect might be expected due to high doses and novelty of our protocol. As observed in the SNT trials, interventional effect fell down from open-trial study to sham-controlled trial [10, 11]. Therefore, the antidepressant effects observed in our study were likely also overestimated.

However, our trial provided insight of high-dose aiTBS application in naturalistic outpatient settings and explored possible maintenance option therapy after such an intensive protocol. We used a modified SNT protocol which was easier to implement in routine clinical practice despite extended hours and suboptimal effect. We tried to enrich the modality of our dataset to provide more understanding of the underlying mechanisms. In future studies the antidepressant effect of accelerated high-dose iTBS protocol should be confirmed through large sham-controlled trials. At the same time, we suggest that an accelerated iTBS protocol with fewer daily sessions, such as 5–8 sessions, could be tried out to reach a balance of accelerated effect and feasibility [50].

## Conclusion

In conclusion, high dosage aiTBS with 18,000 stimuli per day (i.e. 90,000 stimuli in total) was safe, feasible, and associated with higher antidepressant response rates than conventional iTBS protocols [51]. However, aiTBS did not reach the effectiveness of the SNT protocol [10, 11] in this open-label study with the limitations of a small sample and uncontrolled design. Thus, future research may equally focus on precision components (i.e. individualized MRI based targeting and iTBS intensity adjustment) in addition to high dosage aiTBS. 

## Supplementary Information

Below is the link to the electronic supplementary material.

Supplementary file1 (DOCX 742 KB)

## Data Availability

The de-identified individual patient data in this paper will be made accessible after its publication for non-commercial academic projects that have a legitimate research topic and a clearly stated hypothesis. In the event that the application is accepted, researchers will be asked to get the study approved by their institution's ethics board. The authors will subsequently provide the de-identified data sets via a safe data transfer system.
