# Supplementary material for: High dosage accelerated intermittent theta burst stimulation without precision targeting and dosing in depression: an open-label pilot study
Source: Eur Arch Psychiatry Clin Neurosci. 2025 Jul 24;276(2):475–84. doi: 10.1007/s00406-025-02067-z (PMC12953451; doi:10.1007/s00406-025-02067-z)
Supplement: Supplementary file 1 — Supplementary file1 (DOCX 742 KB) [file 406_2025_2067_MOESM1_ESM.docx]

Supplement

**1. BeamF3 measurements and MT determination**

Prior to the first treatment session, BeamF3 measurements and MT determination were performed. We marked BeamF3 coordinates on a cap to calculate the BeamF3 target and then marked coil position and direction for coil placement before each iTBS session. The rMT was determined using an electromyography (EMG) device. The threshold was defined as the lowest intensity that elicits motor-evoked potentials (MEPs) with a minimum amplitude of 0.05 mV from the relaxed thumb and/or index finger muscles in at least 5 out of 10 stimulations of the left motor hotspot region. Before each session, two operators positioned the cap and made sure the coil was positioned with center on the marked BeamF3 target and 45° to the hemispheric midline. The coil was then fixed with a holding arm so that it remained stable throughout the session. Patients were seated in an undisturbed quiet waiting area with separate desks, when it was not their turn. They were allowed to spend the intersessional treatment interval as they preferred but were not allowed to discuss the treatment experiences with each other.

**2. Clinical Scales**

**Montgomery-Asberg Depression Rating Scale (MADRS)**

MADRS was designed to be particularly sensitive to treatment change [1]. This rater-based interview is used to measure the severity of a depressive episode and consists of 10 items.

**Seventeen-item Hamilton Depression Rating Scale (HAM-D)**

Seventeen-item HAM-D is the most widely used clinician-administered depression assessment scale for patient selection and follow-up assessment [2, 3].

**Six-item HAM-D**

Six-item HAM-D is a subscale of 17-item HAM-D, including assessment of mood, feelings of guilt, lassitude, retardation, agitation and somatic symptoms. It is demonstrated to be more valid in measuring the severity of depressive states and sensitive to intervention changes [4]. It was used to assess depressive symptoms after each aiTBS treatment day.

**Beck Depression Inventory (BDI–II)**

BDI-II [5] is a 21-question multiple-choice self-report inventory, one of the most widely used psychometric tests for measuring the severity of depression.

**3. Cognitive functioning assessment**

Cognitive functioning was assessed by the THINC-Integrated Tool (THINC-it®), a user-friendly patient-administered cognitive screening tool that integrates several subjective and objective measures [6, 7]. It comprises variants of four cognitive tests: the Choice Reaction Time Identification Task (CRT, within the app named “Spotter”), One-Back (n-back) Test (NBack, “Symbol Check”), Digit Symbol Substitution Test (DSST, “Code Breaker”) and Trail Making Test-Part B (TMT, “Trails”) . This allows us to test for functions such as attention, working memory, and processing speed [8]. The tool exhibits good psychometric properties and results in the digital version correlating with pen and paper versions of the tasks [6].

**4. Table S1:** Number of failed antidepressant trials, baseline antidepressant medications, baseline non-pharmacotherapy and comorbidities

| ID | Failed antidepressant trials in the current episode* | Baseline ongoing antidepressant medication | Baseline psychotherapy | Previous non-invasive brain stimulation | Comorbidities (M.I.N.I.) |
| --- | --- | --- | --- | --- | --- |
| 1 | Levomilnacipran  Agomelatine  Buproprion  Tianeptine  Lithium | Bupropion 300 mg, Quetiapine 125 mg | - | active tDCS through RCT | Suicidal ideation, panic disorder, social phobia |
| 2 | Duloxetine | Bupropion 150 mg, Fluoxetine 150 mg | Individual CBT | sham tDCS through RCT, outpatient left DLPFC iTBS treatment | Suicidal ideation and suicidal behavior disorder, post -traumatic stress disorder |
| 3 | Citalopram | Citalopram 20 mg, Mitrazapine 30 mg | Behavior therapy since 2021 | active tDCS through RCT | Panic disorder, social phobia |
| 4 | - | - | Individual CBT | active tDCS through RCT | - |
| 5 | Duloxetine  Buproprion | Dulexetine 120 mg, Bupropion 300 mg, Lithium 1200 mg, Quetiapine 50 mg | Psychotherapy once a month | outpatient left DLPFC iTBS treatment | - |
| 6 | Venlafaxine | Venlafaxine 150 mg, Quetiapine 50 mg | Outpatient psychotherapy since 2018, behavior therapy since 2023 | - | Generalized anxiety disorder, suspected trauma |
| 7 | Venlafaxine  Mitrazapine | Venlafaxine 150 mg | Group psychotherapy | bilateral iTBS through RCT, condition not revealed | Panic disorder |
| 8 | Fluoxetine | Fluoxetine 60 mg | - | - | - |

*According to ATHF-SF.

**5. Table S2: Medication and non-pharmacotherapy during 6 months after the high-dose aiTBS**

| Patient ID | Antidepressant medication | Non-invasive brain stimulation |
| --- | --- | --- |
| 1 | Tranylcypromine 30 mg, Quetiapin at night 100 mg | 5 weeks clinical tDCS |
| 2 | Fluoxetine 150 mg | - |
| 3 | Sertraline 50 mg, Bupropion | 6 weeks clinical left DLPFC standard iTBS (3 weeks daily, 3 weeks twice per week) |
| 4 | Bupropion 300 mg, Risperidone 0.5 mg | - |
| 5 | lost FU | lost FU |
| 6 | Venlafaxine, Quetiapine, Lorazepam only for emergency | - |
| 7 | Mirtazapine 15 mg | - |
| 8 | lost FU | lost FU |

**6. Table S3: Side effects of high-dose accelerated iTBS**

| Type | Percentage |
| --- | --- |
| Fatigue | 87.5% (7) |
| Sleep disturbance | 75% (6) |
| Gastroenterological problem | 25% (2) |
| Muscle twitch | 12.5% (1) |
| Migraine | 12.5% (1) |
| Euphoria experience | 12.5% (1) |
| Subtle resting/postural tremors | 12.5% (1) |

Two patients experienced gastrointestinal problems but they were not believed to be triggered by the stimulation due to the frequent occurrence in past life or suspected infection through family members. One patient had muscle twitch in the left arm, but it did not cause any inconvenience to everyday life. One patient with a history of migraine attacks had quite intense migraine and had to take a rest on the third day, but continued the treatment after one day break. One patient had extreme euphoria from the second treatment day on, but this state distinguished after the weekend. One patient had subtle resting/postural tremors on the fifth treatment day, which has not been reported by any previous iTBS trial.

**7. Table S4: Score change, response and remission rate after accelerated high-dose iTBS treatment**

| **Timepoint** | **Post-treatment** | **Follow-up 1** | **Follow-up 2** | **Follow-up 3** | **Follow-up 4** | **post-tDCS** | **6-month follow-up** |
| --- | --- | --- | --- | --- | --- | --- | --- |
| MADRS score change, mean(SD) | -13.13 (7.84) | -11.5 (5.62) | -14.003 (5.54) | -14.25 (7.85) | -12.50 (9.81) | -10.13 (8.85) | -9.96 (5.71) |
| MADRS response rate | 50.00% | 25.00% | 37.50% | 37.50% | 50.00% | 28.57% | 16.67% |
| MADRS remission rate | 37.50% | 25.00% | 12.50% | 25.00% | 12.50% | 14.28% | 0% |
| 17-item HAM-D score change, mean (SD) | -10.75 (4.79) | -9.31 (3.55) | -10.88 (4.63) | -11.38 (5.32) | -10.38 (6.07) | -9.02 (6.87) | -7.55 (4.23) |
| 17-item HAM-D response rate | 62.50% | 37.50% | 62.50% | 37.50% | 75.00% | 42.86% | 33.33% |
| 17-item HAM-D remission rate | 37.50% | 25.00% | 37.50% | 37.50% | 25.00% | 28.57% | 16.67% |
| BDI-II score change, mean (SD) | -10.34 (10.90) | -4.63 (7.77) | -6.34 (8.50) | -10.92 (11.77) | -8.20 (10.37) | -7.77 (12.17) | -4.96 (12.44) |
| BDI-II response rate | 14.29% | 0% | 14.29% | 28.57% | 14.29% | 28.57% | 33.33% |
| BDI remission rate | 14.29% | 0% | 0% | 14.29% | 14.29% | 14.28% | 16.67% |
| 6-item HAM-D score change, mean (SD) | -6.50  (2.85) | -5.52  (2.48) | -6.63  (2.31) | -6.75  (2.97) | -7.13  (2.66) | -5.81  (3.60) | -5.55  (1.72) |
| 6-item HAM-D response rate | 50.00% | 57.14% | 62.50% | 50.00% | 75.00% | 42.86% | 50.00% |
| 6-item HAM-D remission rate | 37.50% | 28.57% | 37.50% | 25.00% | 37.50% | 42.86% | 0% |

8. **Fig. S1: 6-month clinical outcome**


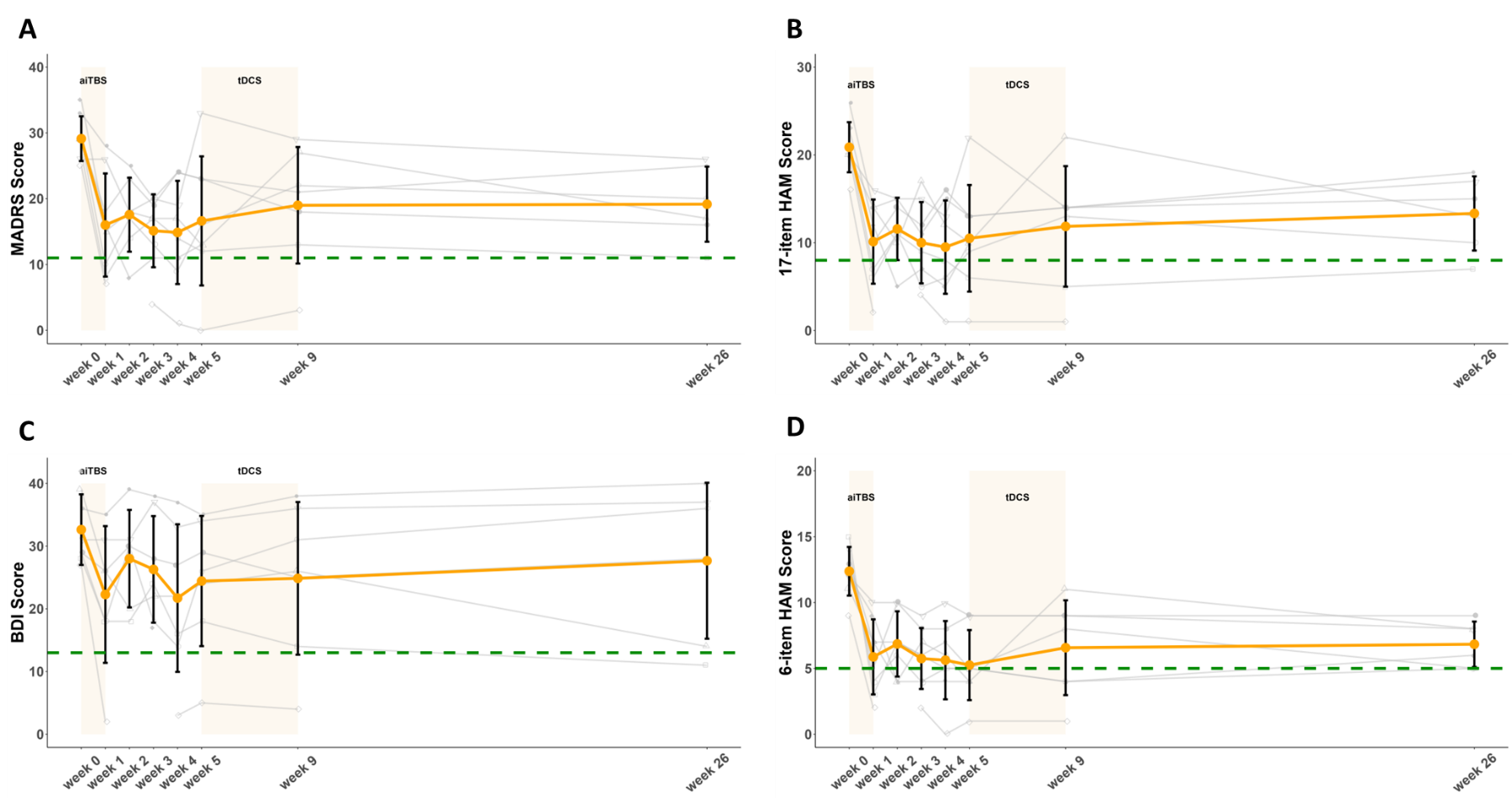
Week 0 = Baseline, Week 1 = post-treatment, Week 2 = FU 1, Week 3 = FU 2, Week 4 = FU 3, Week 5 = FU 4, Week 9 = post-tDCS, Week 26 = 6-month FU. Panel A depicts the MADRS score change, with remission defined as a score < 11. Panel B depicts the 17-item HAM-D change, with remission defined as a score < 8. Panel C depicts the BDI-II change, with remission defined as a score < 13. Panel D depicts the daily 6-item HAM-D change, with remission defined as a score < 5. Remission thresholds are indicated by green dashed lines. Individual trajectories are represented by grey lines. The gaps in the line plots correspond to missing values, either due to missing treatment sessions or missed follow-up visits.

**9. Fig. S2: MADRS item-wise change**

**10. Fig. S3: 6-item HAM-D item-wise change**
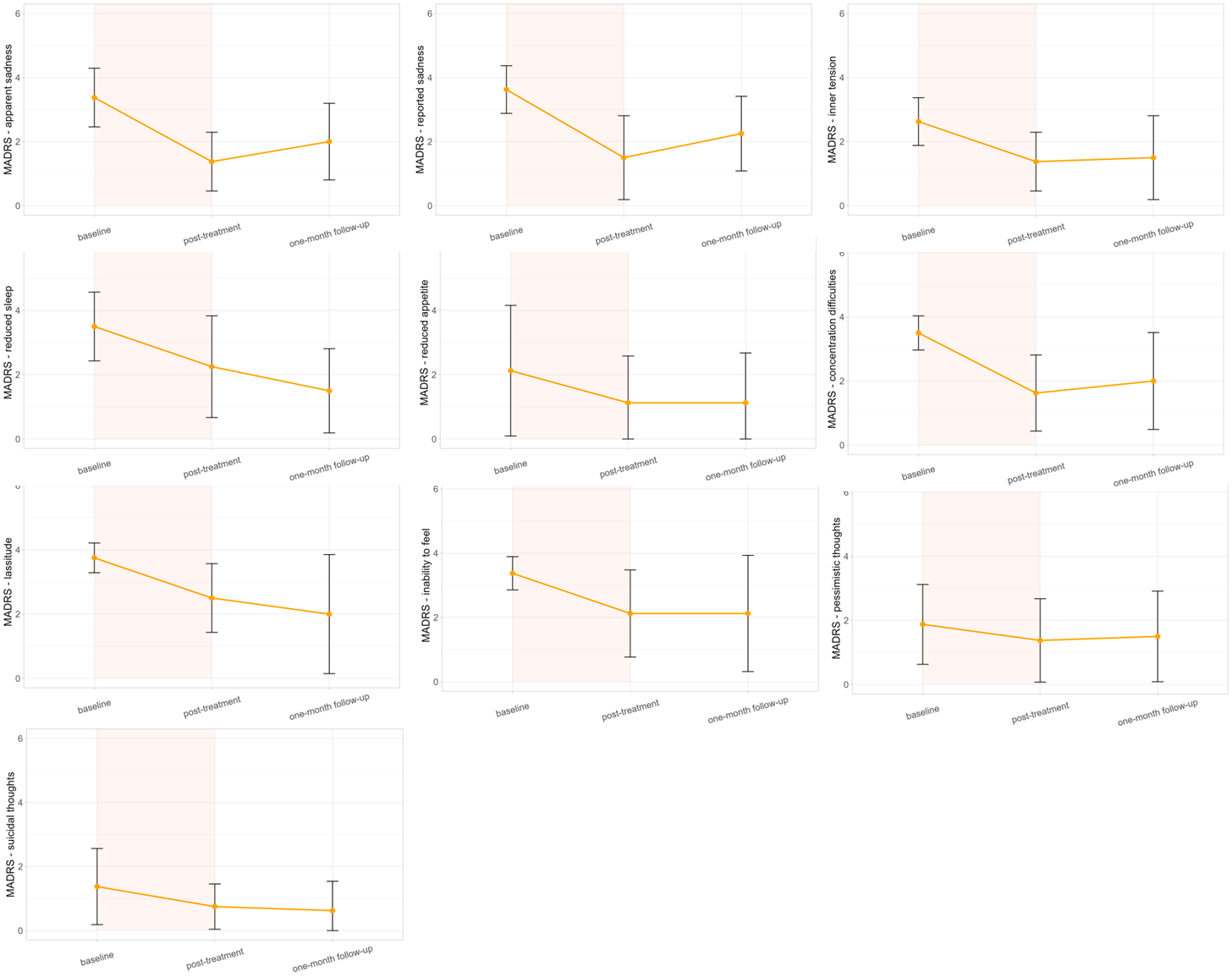


**
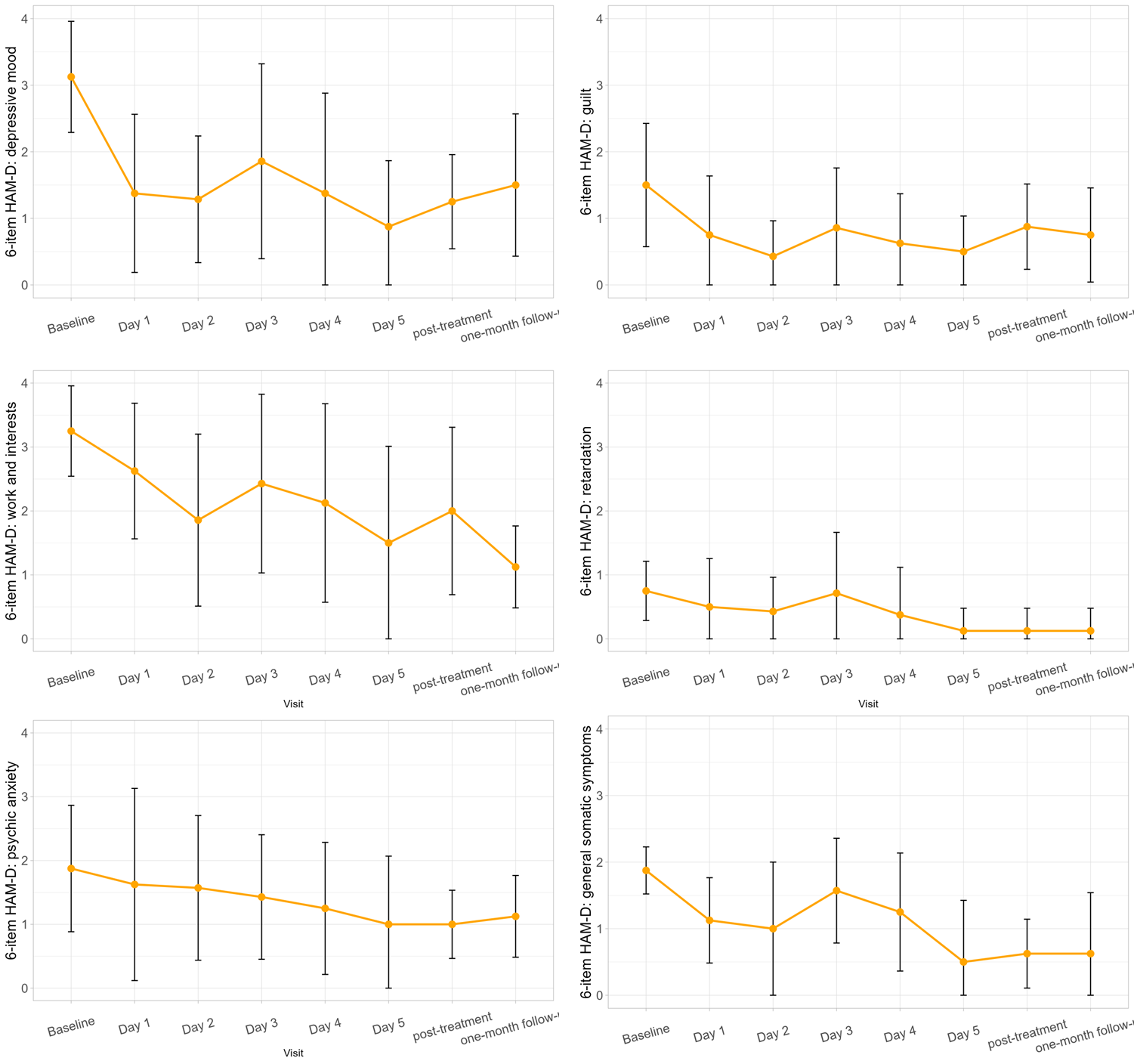
**

**11. Table S5: tDCS maintenance therapy regime**

| Patient | Start | End | tDCS session |
| --- | --- | --- | --- |
| 1 | 14.11.2023 | 14.12.2023 | 13 |
| 2 | 16.11.2023 | 16.12.2023 | 14 |
| 3 | 16.11.2023 | 09.01.2024 | 33 |
| 4 | 16.11.2023 | 14.12.2023 | 3 |
| 5 | 28.12.2023 | 06.02.2024 | 9 |
| 6 | 28.12.2023 | 31.01.2024 | 10 |
| 7 | 06.02.2024 | 19.03.2024 | 15 |
| 8 | 17.01.2024 | 19.02.2024 | 9 |

**Supplement references**

1. Montgomery SA, Åsberg M (1979) A New Depression Scale Designed to be Sensitive to Change. Br J Psychiatry 134:382–389. https://doi.org/10.1192/bjp.134.4.382

2. Hamilton M (1967) Development of a Rating Scale for Primary Depressive Illness. Br J Soc Clin Psychol 6:278–296. https://doi.org/10.1111/j.2044-8260.1967.tb00530.x

3. Williams JBW (1988) A Structured Interview Guide for the Hamilton Depression Rating Scale. Arch Gen Psychiatry 45:742. https://doi.org/10.1001/archpsyc.1988.01800320058007

4. Bech P, Gram LF, Dein E, et al (1975) QUANTITATIVE RATING OF DEPRESSIVE STATES: Correlation Between Clinical Assessment, Beck’s Self‐Rating Scale and Hamilton’s Objective Rating Scale. Acta Psychiatr Scand 51:161–170. https://doi.org/10.1111/j.1600-0447.1975.tb00002.x

5. Beck, A. T., Steer, R. A., & Brown, G. (1996) Beck Depression Inventory–II (BDI-II) [Database record]. APA PsycTests

6. McIntyre RS, Best MW, Bowie CR, et al. (2017) The THINC-Integrated Tool (THINC-it) Screening Assessment for Cognitive Dysfunction: Validation in Patients With Major Depressive Disorder. J Clin Psychiatry 78:873–881. https://doi.org/doi:10.4088/JCP.16m11329

7. Culpepper L, Lam RW, McIntyre RS (2017) Cognitive Impairment in Patients With Depression: Awareness, Assessment, and Management: (Academic Highlights). J Clin Psychiatry 78:1383–1394. https://doi.org/10.4088/JCP.tk16043ah5c

8. Baune BT, Malhi GS, Morris G, et al (2018) Cognition in depression: Can we THINC-it better? Journal of Affective Disorders 225:559–562. https://doi.org/10.1016/j.jad.2017.08.080
